# Cryopreservation method for *Entamoeba histolytica* trophozoites

**DOI:** 10.1128/msphere.00889-25

**Published:** 2026-03-30

**Authors:** Makoto Kazama, Fumika Mi-ichi

**Affiliations:** 1 NEKKEN Bio-Resource Center, Institute of Tropical Medicine (NEKKEN), Nagasaki University, Nagasaki, Japan; 2 Central Laboratory, Institute of Tropical Medicine (NEKKEN), Nagasaki University, Nagasaki, Japan; University of California Davis, Davis, California, USA

**Keywords:** cryopreservation, *Entamoeba histolytica*, revival rate, cooling rate

## Abstract

*Entamoeba histolytica*, a protozoan parasite, causes amebiasis, which is a global public health problem. Clinical manifestation and pathogenesis of amebiasis are closely associated with the proliferation and tissue invasion abilities of *E. histolytica* trophozoites. At the same time, *E. histolytica* trophozoites are studied in a broad range of biology research fields. As such, the development of *in vitro* culture of *E. histolytica* trophozoites greatly facilitated amebic research. Now, a standard method for cryopreservation of *E. histolytica* trophozoites is required because available methods either do not give a high enough revival rate, which can significantly delay projects, or they are not widely adopted. Here, we attempted to optimize the conditions for *E. histolytica* trophozoite cryopreservation, including cell density, cooling rate, cell freezing reagent, and freezing profile. We found an optimized condition that reproducibly yielded >30% revival, regardless of storage period in liquid nitrogen (up to 365 days). This optimized condition is that *E. histolytica* trophozoites are suspended in 0.5 mL CELLBANKER 2 in a 1 mL cryotube at 2 × 10^6^ cells/mL and frozen from 4 to −40°C at a rate of −0.2°C/min using VIA Freeze Uno. This cryopreservation method can minimize the risk of losing *E. histolytica* trophozoite lines required to continue projects, facilitating amebic research.

**IMPORTANCE:**

Amebiasis, which is caused by *Entamoeba histolytica* infection, is the third deadliest parasitic disease globally. Proliferation of *E. histolytica* trophozoites and their invasion into the host tissues cause amebiasis symptoms and pathogenesis. *E. histolytica* trophozoites are also important in multiple biology research topics. Therefore, *E. histolytica* trophozoites are a common subject in academic as well as clinical fields. A standard method for *in vitro* culture of *E. histolytica* trophozoites is well established. By contrast, a widely adopted practical method for cryopreservation of *E. histolytica* trophozoites is not yet available. This hampers the advancement of amebic research, as the required *E. histolytica* trophozoite lines sometimes cannot be revived from cryopreservation. In this study, we varied parameters critical to the revival rate, namely, cell density, cooling rate, freezing reagent, and freezing profile, and present an optimized cryopreservation method for *E. histolytica* trophozoites, which gives reproducibly high revival rates.

## INTRODUCTION

Amebiasis is caused by the infection of *Entamoeba histolytica*, a protozoan parasite belonging to the Phylum Amoebozoa. Amebiasis is the third deadliest parasitic disease worldwide (1). However, available drugs are limited, and effective vaccines are not developed yet; therefore, amebiasis is a global public health problem. Diarrhea is a typical clinical sign of amebiasis, and this can develop into amebic dysentery and abscesses, with liver abscesses being most common. These clinical manifestations of amebiasis are closely associated with trophozoite proliferation of *E. histolytica*. Trophozoites, which hatch from orally ingested cysts in the small intestine, move to the large intestine and proliferate there. Some proliferating trophozoites adhere to and kill colonic epithelial cells, leading to dysentery. Trophozoites also secrete proteases that degrade the extracellular matrix and permit invasion into the intestinal wall and beyond (2, 3). Trophozoites that invade the intestinal mucosa can disseminate hematogenously, most commonly causing liver abscesses via the portal venous circulation (4). *E. histolytica* trophozoites are, therefore, the principal target for the development of new drugs and vaccines against amebiasis. Accordingly, an in-depth biochemical and physiological analysis of trophozoites is required.

*E. histolytica* trophozoites are also relevant to a broad range of biological research fields, such as the central dogma of molecular biology, membrane trafficking, cell-cell communication, host-parasite interaction, signal transduction, metabolism, and mitochondrial evolution (5–10). We briefly highlight a few such examples. *E. histolytica* trophozoites perform trogocytosis, a typical biological process in which one cell physically ingests the cellular material from another cell via cell-cell interaction. Although trogocytosis is well studied in immune cells and also found in other types of cells (11, 12), *E. histolytica* trophozoites perform trogocytosis not only to kill the host cells but also to acquire human cell membrane proteins and display them on the cell surface, which protects them from cell lysis by the host complement system (13). Accordingly, trogocytosis is important to the pathogenesis of *E. histolytica* infections (14, 15). *E. histolytica* also possesses highly diversified mitochondria, which are called mitosomes. This organelle lacks almost all functions present in aerobic mitochondria, such as oxidative phosphorylation and *β*-oxidation of fatty acids (5, 16). However, mitosomes retain the sulfate activation pathway that is essential for the *Entamoeba* life cycle (7, 17–19). This feature is unexpected because the sulfate activation pathway typically functions in the cytoplasm or plastids (20, 21). Many unique features have been unraveled to date, and still more features remain to be discovered in *Entamoeba* parasites.

Given not only the clinical requirements for new drugs and vaccines against amebiasis but also the academic interests, maintenance of live *E. histolytica* trophozoites is indispensable. The development of *in vitro* culture of *E. histolytica* trophozoites (22) contributed enormously to the advancement of amebic research. Currently, the establishment of a standard method for cryopreservation of trophozoites is required for the progress of such research. To that end, several methods have been reported (23–27); however, a method that is widely adopted and provides sufficiently high revival rates following cryopreservation has not been established yet. These circumstances cause problems for researchers that may be forced to maintain very long-term *in vitro* cultures and for projects that are significantly delayed due to the loss of *E. histolytica* trophozoite lines. In this study, we attempted to establish an optimized method for cryopreservation of *E. histolytica* trophozoites by using an easy, rate-controllable freezing machine together with commercially available cell freezing reagents and cryotubes, with which a monitor strain, *E. histolytica* HM-1:IMSS, was reproducibly revived with a high rate (34.6 ± 5.7%).

## MATERIALS AND METHODS

### *E. histolytica* strain and *in vitro* culture

*E. histolytica* HM-1:IMSS cl6 was provided by Dr. Seiki Kobayashi (Japan Institute for Health Security) via the National Bio Resource Project (NBRP), which was supported by MEXT of Japan. The trophozoites were routinely maintained under anaerobic culture conditions using BI-S-33 medium (22) containing 15% adult bovine serum and antibiotics (1 U/mL penicillin and 0.1 mg/mL streptomycin) in screw-capped glass tubes or 25 cm^2^ culture flasks at 36.5°C.

### Cell freezing reagents and machines for preparing cryopreservation

CELLBANKER 1 and 2 were purchased from ZENOGEN PHARMA Co., Ltd. (Fukushima, Japan). NAGASE’s cryopreservation solution-1 was from NAGASE & Co., Ltd. (Kyoto, Japan). Synth-a-Freeze cryopreservation medium was from Thermo Fisher Scientific K.K. (Tokyo, Japan). COS banker and -II were from COSMO BIO Co., Ltd. (Tokyo, Japan).

BICELL, a freezing container, and VIA Freeze Uno, a rate-controllable freezing machine, were purchased from NIHON FREEZER Co., Ltd. (Tokyo, Japan) and Cytiva (Tokyo, Japan), respectively.

### Preparation of cryopreservation samples of *E. histolytica* trophozoites

A widely adopted method was used as the basis to optimize the cryopreservation conditions for *E. histolytica* trophozoites (26) with some modifications. In detail, 0.5 × 10^6^ cells of *E. histolytica* trophozoites were pelleted by centrifugation at 500 × *g* for 5 min at 4°C and suspended in 0.5 mL CELLBANKER 2 and transferred into a 1.0 mL cryotube (Nunc CryoTube 375,353; Thermo Fisher Scientific). Cell densities in routine cultures of *E. histolytica* trophozoites were determined by manual counting under a microscope using a Fuchs-Rosenthal’s hemocytometer (Sunlead Glass Co., Ltd., Saitama, Japan). Cryotubes containing cell suspensions were kept in a freezing container, BICELL (NIHON FREEZER Co., Ltd.), and then stored at −80°C overnight. On the following day, cryotubes were transferred to a liquid nitrogen tank (LS750; IC Biomedical, LLC, GA USA).

### Statistics

Statistical analyses were performed using Student’s *t*-tests.

### Determination of revival rate

After storage in liquid nitrogen for the testing period, cryotubes containing *E. histolytica* trophozoite cell suspensions were warmed by hand until frozen solutions were completely melted. The suspensions were added to 6 mL YIMDHA-S medium (28). After gentle mixing, a portion was used to determine the revival rate (%), which is the ratio of trypan-blue unstained cell number to the total cell number as described previously (29). The trypan-blue unstained cells were considered to be successfully revived. Trypan-blue unstained cell numbers and total cell numbers were manually counted under a microscope.

### Optimization of freezing conditions

To find an optimum cell density for cryopreservation, different cell numbers of *E. histolytica* trophozoites (0.5, 1.0, 1.5, 2.0, 3.0, 4.0, and 8.0 × 10^6^ cells) were suspended in 0.5 mL CELLBANKER 2 (ZENOGEN PHARMA Co., Ltd.) using a 1 mL cryotube. The cryotubes were frozen at a cooling rate of −1.0°C/min using VIA Freeze Uno (Cytiva). Immediately after the program was finished, the cryotubes were transferred to BICELL (NIHON FREEZER Co., Ltd.), a freezing container and stored at −80°C overnight. Then, the cryotubes were stored in liquid nitrogen for the testing period. Determination of the revival rates was performed as described above, and the best cell density was determined.

To determine an optimum cooling rate, different rates (−0.02, −0.05, −0.1, −0.2, −0.5, −0.75, −1.0, −1.2, −1.5, and −2 °C/min) from 4 to −40°C were set to freeze *E. histolytica* trophozoites suspended in CELLBANKER 2 (0.5 mL each in a 1 mL cryotube) using VIA Freeze Uno (Cytiva). Below −40°C, the cooling rate was kept at −2°C/min until reaching −100°C. Final freezing, storage, and determination of the revival rates were performed as described above, and the best cooling rate was determined.

To select the best cell freezing reagent, the six reagents were used to suspend *E. histolytica* trophozoites at 2.0 × 10^6^ cells/mL, and 0.5 mL of each cell suspension was dispensed into cryotubes. The cryotubes were frozen at the best rate as determined above using VIA Freeze Uno (Cytiva). Final freezing, storage, and determination of the revival rates were performed as described above, and the best cell freezing reagent was determined.

## RESULTS

### Optimizing cryopreservation conditions including cell density, cooling rate, freezing reagent, and freezing program

To optimize cryopreservation conditions for *E. histolytica* trophozoites, four parameters that have been assumed to have significant impact on the revival rate were varied and assessed by the cell viability. In all analyses, a VIA Freeze Uno (Cytiva), a controlled-rate freezing machine, was used. We selected the VIA Freeze Uno from among commercially available controlled-rate freezers because it allows the slowest cooling rate of −0.02°C/min. A pilot condition was set in which *E. histolytica* trophozoites were suspended in CELLBANKER 2 at a density of 1.0 × 10^6^ cells/mL (0.5 mL in a 1 mL cryotube), and a cooling rate of −1°C/min was used. This pilot condition reproducibly yielded ~5% revival rates regardless of the storage period in liquid nitrogen.

#### Cell density

To find an optimum cell density, only cell density was changed in the above pilot condition. Among the seven different densities tested (0.5–8 × 10^6^ cells/mL), 2 × 10^6^ cells/mL gave the highest revival rate at ~10%; however, there were no statistically significant differences (Fig. S1).

#### Cooling rate

**Fig 1:**
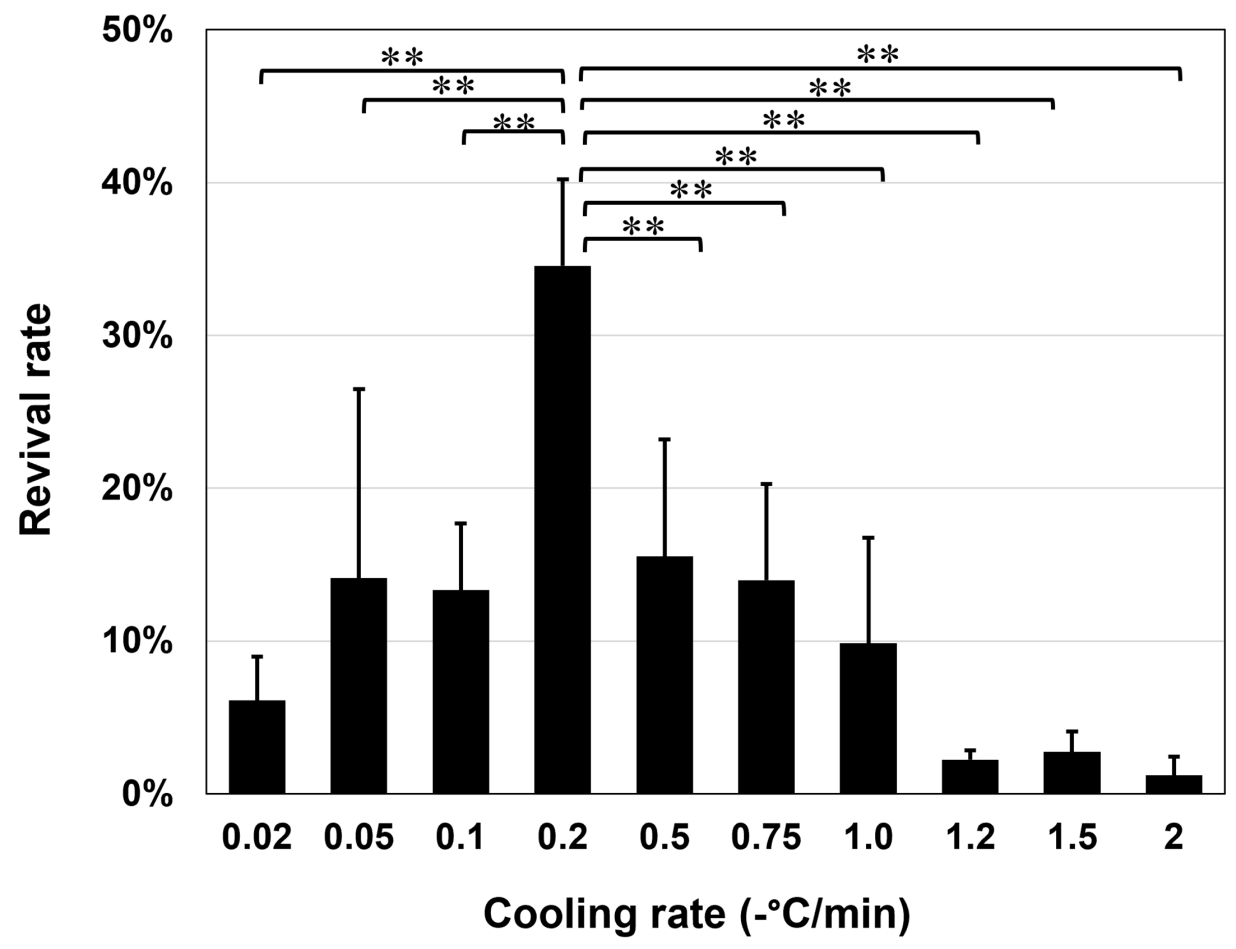
Effect of cooling rate on the revival rate of *E*. *histolytica* trophozoite cells. Ten different cooling rates were set from 4°C until −40°C. In the temperature range above 4°C and below −40°C, the cooling rate was set at −2.0°C/min. Cell densities were adjusted to 2.0 × 10^6^ cells/mL in 0.5 mL CELLBANKER 2 in a 1 mL cryotube. Revival rates were calculated by trypan blue staining after storage in a liquid nitrogen tank for 6 days. The data are represented as mean ± SD of nine samples from two independent experiments. Statistical significance was assessed by an unpaired two-tailed *t*-test versus−0.2°C/min cooling rate condition. **P < 0.01.

**Fig 2:**
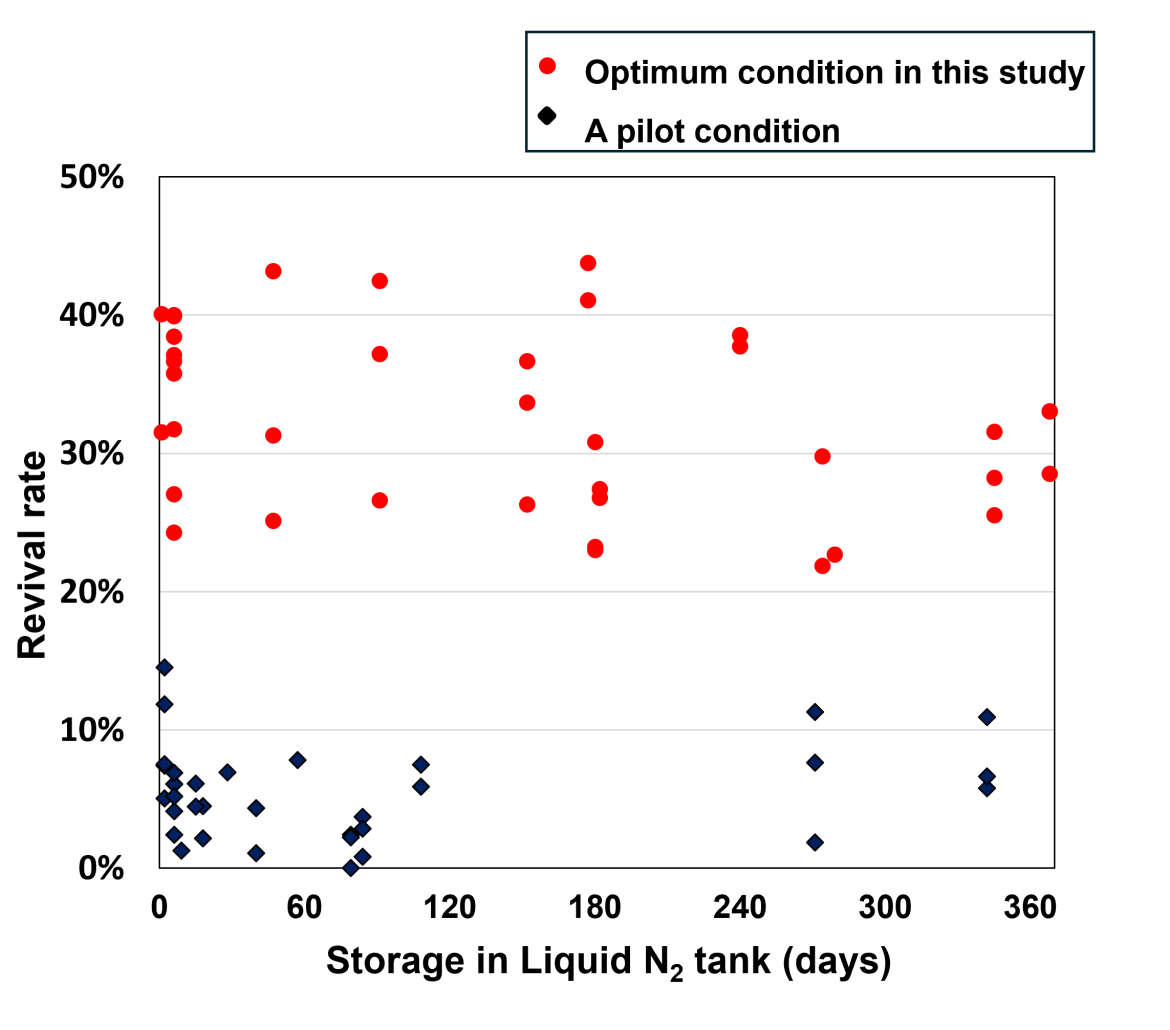
Effect of storage period in liquid nitrogen tank on revival rate of *E*. *histolytica* trophozoite cells. Revival rates after different lengths of storage in the liquid nitrogen tank were determined by trypan blue staining. The cryopreserved conditions were set at 2.0 × 10^6^ cells/mL in 0.5 mL CELLBANKER 2 in a 1 mL cryotube and −0.2°C/min cooling rate in the range from 4°C to −40°C (optimum condition in this study; red circles), and at 1.0 × 10^6^ cells/mL in 0.5 mL CELLBANKER 2 in a 1 mL cryotube and −1.0°C/min cooling rate in the range from 4 to −40°C (a pilot condition set in this study; black diamonds). The revival rate for the latter was not different from that of samples frozen by a method with BICELL (6.9 ± 2.2%, *n* = 3, *P* > 0.05).

Subsequently, at above optimized cell density (2 × 10^6^ cells/mL), the cooling rates from 4 to −40°C were varied (−0.02 to −2.0°C/min). Among the 10 different rates, the −0.2°C/min cooling rate gave the best revival rate at 34.6 ± 5.7% (Fig. 1). Furthermore, across the liquid nitrogen storage periods tested (up to 365 days), the −0.2°C/min cooling rate reproducibly gave much higher revival rates than a pilot condition, in which cell density was 1 × 10^6^ cells/mL, and the cooling rate was −1.0°C/min (Fig. 2). These values were the same as those in previous studies (26, 27). This improvement with optimized cryopreservation conditions, in which *E. histolytica* trophozoites were suspended in 0.5 mL CELLBANKER 2 in a 1 mL cryotube (final cell density, 2 × 10^6^ cells/mL) with freezing at −0.2°C/min from 4 to −40°C using VIA Freeze Uno (Cytiva), suggests that they can be stored long-term in liquid nitrogen, perhaps indefinitely.

#### Freezing reagent

**Fig 3:**
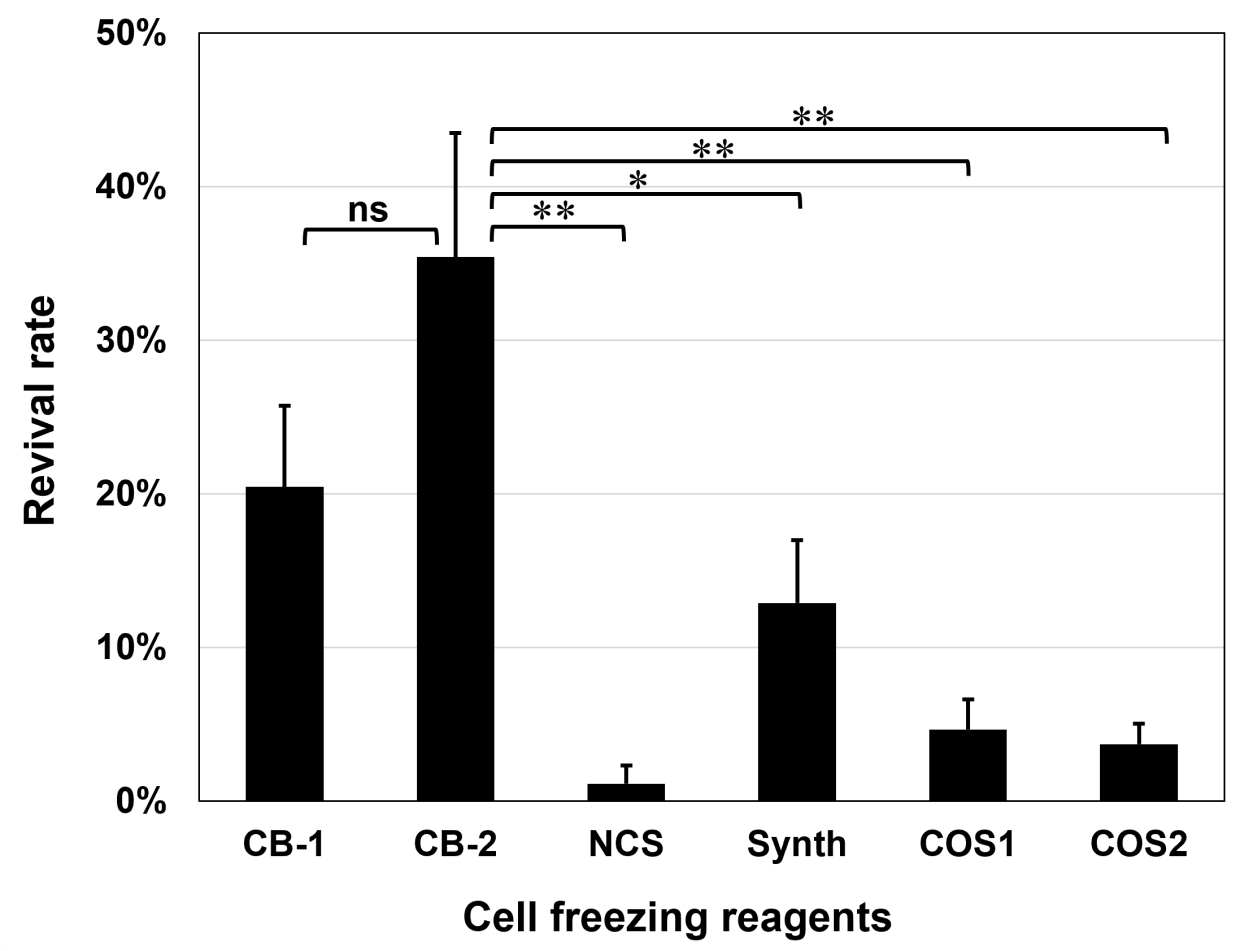
Comparison of commercially available cryopreservation solutions for the revival rate of *E*. *histolytica* trophozoite cells. Six cell freezing reagents (CB-1, CELLBANKER 1; CB-2, CELLBANKER 2; NCS, Nagase’s Cryopreservation Solution 1; Synth, Synth-a freeze; COS1, COSBANKER; COS2, COSBANKER-II) were used. The cryopreservation conditions were the same as the red plots in Fig. 2. Revival rates were determined by trypan blue staining after storage in a liquid nitrogen tank for 6 days. The data are represented as mean ± SD of six samples from two independent experiments. *P < 0.05; **P < 0.01; ns, not significant.

Next, six commercially available cell freezing reagents were tested with the above optimized cell density and cooling rate. Among the six reagents, CELLBANKER 2 (ZENOGEN PHARMA Co., Ltd.) gave the highest revival rate at 35.4 ± 8.1% (Fig. 3).

#### Freezing program

**Fig 4:**
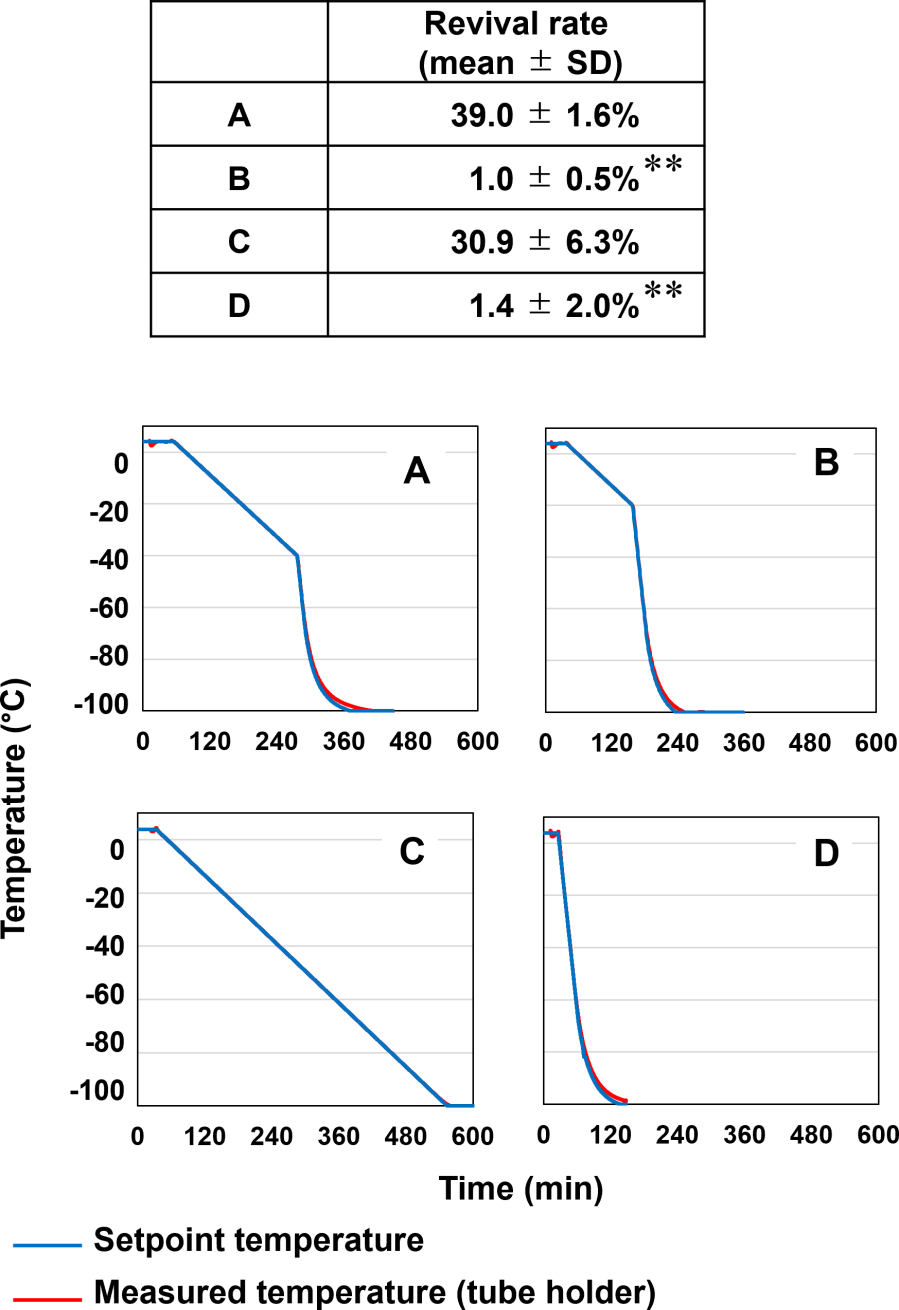
Effect of four freezing profiles on revival rate of *E*. *histolytica* trophozoite cells. At cooling rates of −0.2°C/min within different temperature ranges (**A–C**) and −2.0°C/min (**D**), revival rates were determined by trypan blue staining. The cell density was fixed at 2.0 × 10^6^ cells/mL in 0.5 mL CELLBANKER 2 in a 1 mL cryotube, and cryotubes were stored in liquid nitrogen for 6 days. Values are presented as mean ± SD (*n* = 3). Statistical significance was assessed by an unpaired two-tailed *t*-test versus A. **P < 0.01. Setpoint temperature (blue lines) and measured temperature in the tube holder (red lines) are shown.

Finally, the cooling program endpoint was varied to find the optimum temperature before freezing in liquid nitrogen. Cooling at −0.2°C/min until −40°C yielded a much higher revival rate (39 ± 1.6%) than cooling until −20°C (1.0 ± 0.5%) (Fig. 4A and B). Interestingly, when this cooling program was extended to reach −100°C, the revival rate was not significantly changed (39 ± 1.6% vs 30.9 ± 6.3%) (Fig. 4A and C). As shown previously (Fig. 1), when the cooling rate was elevated to −2.0°C/min, the revival rate significantly reduced (1.4 ± 2.0%), even when the final temperature was set at −100°C (Fig. 4D). These results show that an optimum cooling rate (−0.2°C/min) must be maintained until samples reach −40°C.

Taken together, the optimum cryopreservation conditions that reproducibly give a much higher revival rate than a pilot condition set in this study (>30% [present study] vs <10%) for *E. histolytica* trophozoites is suspension in 0.5 mL CELLBANKER 2 in a 1 mL cryotube (final cell density, 2 × 10^6^ cells/mL) with freezing at −0.2°C/min from 4 to −40°C or colder using an easy rate-controllable freezer, VIA Freeze Uno (Cytiva).

## DISCUSSION

Establishment of a standard method for cryopreservation of *E. histolytica* trophozoites is required because revival rates from cryopreservation, as obtained by available methods, are not sufficient to facilitate academic as well as clinical *E. histolytica* studies. In this study, we focused on four factors that have been assumed to have significant impact on revival rate, namely, cell density, cooling rate, freezing reagent, and freezing profile.

Albeit not statistically significant, 2 × 10^6^ cells/mL (0.5 mL cell suspension in a 1 mL cryotube) constantly yielded the highest revival rate when freezing at a rate of −1°C/min using a rate-controllable freezer, VIA Freeze Uno (Cytiva). Importantly, this programmable freezing machine enabled optimization of the cooling rate to −0.2°C/min by testing a wide range of rates. This same freezing machine was also used to compare cell freezing reagents, with CELLBANKER 2 (ZENOGEN PHARMA Co., Ltd.) being the best at the tested condition (cell density, 2 × 10^6^ cells/mL [0.5 mL cell suspension in a 1 mL cryotube]; cooling rate, −0.2°C/min).

Various freezing containers and rate-controllable freezing machines that are commonly used and commercially available are used with a cooling rate of −1.0°C/min. This cooling rate gives high revival rates and/or viability for cryopreserved samples of many parasitic protozoa, including the trophozoites of *Giardia lamblia* (~84%) (30, 31) and *Trichomonas vaginalis* (73 ± 8%) (32), tachyzoites of *Toxoplasma gondii* (95 ± 4%) (33), gametocytes of *Plasmodium falciparum* (40 ± 5%) (34), trypomastigotes of *Trypanosoma cruzi* (~20%) (35), and blood stream forms of *Trypanosoma brucei* (~60%) (36). In the case of *E. histolytica trophozoites*, however, the much lower cooling rate of −0.2°C/min was optimal (>30%), revealing that the cooling rate is a critical factor significantly affecting the revival rate of trophozoites. In addition, the optimal range of the cooling rate for *E. histolytica trophozoite* cryopreservation is very narrow, as changing to −0.1 and −0.5°C/min drastically decreased revival rates.

Previously, several studies on the relationship between the cooling rate or cooling temperature range and the revival rate (viability) following cryopreservation were published (23, 27). Of note, Diamond (27) suggests that the latent heat of fusion zone (LHFZ) is an important factor for the success of cryopreservation. The LHFZ is the heat energy that is released when a liquid becomes a solid without changing its temperature. Diamond’s most successful protocol, in which the cooling cycle was designed with consideration for the LHFZ, was: −8 to −10°C/min from room temperature to 2°C (above the LHFZ), passing through the LHFZ as quickly as possible, followed by −1 to −2°C/min from the LHFZ down to −40°C. His assumption is supported by our observation that an optimum cooling rate must be maintained from 4 to −40°C, which includes the LHFZ for an aqueous solution.

This study showed that a slow cooling rate (−0.2°C/min) within a narrow range is critical for *E. histolytica* trophozoite cryopreservation as compared with those for other protozoan species (−1°C/min). An explanation for this difference may be the structure and composition of lipids in the *E. histolytica* trophozoite cellular membrane, particularly the sphingolipids (SLs) and glycerophospholipids (GPLs) (37). SLs and GPLs are major components of the cellular membrane, and they affect the sensitivity to temperature-dependent changes in the membrane rigidity and fluidity (38–40). In *Entamoeba* trophozites, these lipid classes atypically and dominantly have saturated, mono-unsaturated, or di-unsaturated very long (26–30 carbons) acyl chains and/or saturated medium (8–12 carbons) acyl chains (37, 41). Therefore, atypical lipid structure and composition in *Entamoeba* cell membrane may be a reason to require a slow cooling rate within a narrow range for cryopreservation.

Finally, we present a new method for cryopreservation of *E. histolytica* trophozoites, from which ~35% revival rate is reproducibly achieved. This revival rate of *E. histolytica* trophozoite cells has a significant impact on subsequent *in vitro* cultures, facilitating clinical as well as academic *E. histolytica* studies.

## SUPPLEMENTAL MATERIAL

Fig. S1 msphere.00889-25-s0001.tif Pilot experiment for determining optimum cell density for cryopreserved stocks of *E. histolytica* trophozoites.

ASM does not own the copyrights to Supplemental Material that may be linked to, or accessed through, an article. The authors have granted ASM a non-exclusive, world-wide license to publish the Supplemental Material files. Please contact the corresponding author directly for reuse.
