# Supplementary figures and images for: Cryopreservation method for Entamoeba histolytica trophozoites
Source: mSphere. 2026 Mar 30;11(4):e00889-25. doi: 10.1128/msphere.00889-25 (PMC13123703; doi:10.1128/msphere.00889-25)

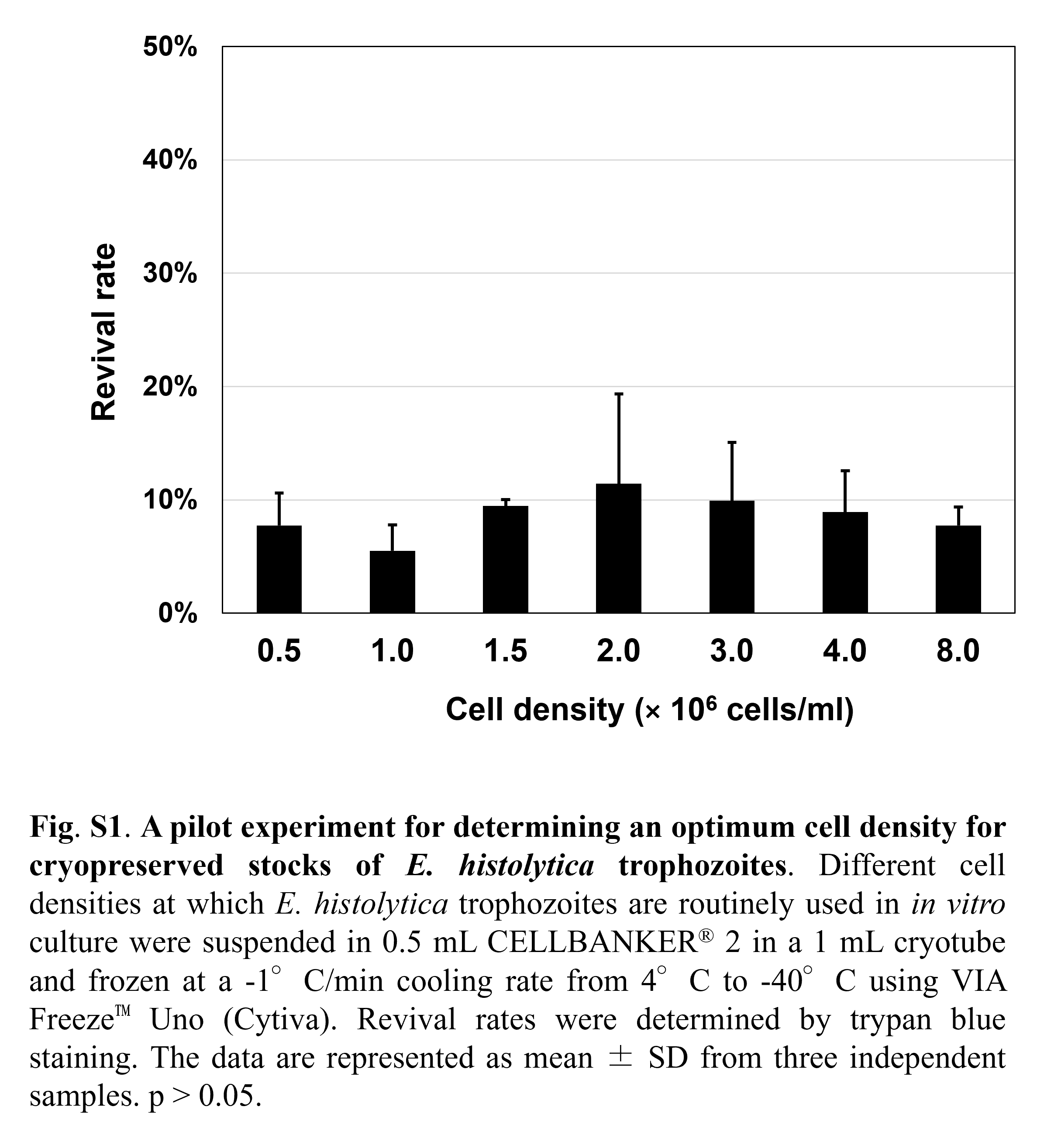

Supplement: Fig. S1 — Pilot experiment for determining optimum cell density for cryopreserved stocks of E. histolytica trophozoites. [file msphere.00889-25-s0001.tif]
